# The role of nitric oxide on rosuvastatin-mediated S-nitrosylation and translational proteomes in human umbilical vein endothelial cells

**DOI:** 10.1186/1477-5956-10-43

**Published:** 2012-07-16

**Authors:** Bin Huang, Fu An Li, Chien Hsing Wu, Danny Ling Wang

**Affiliations:** 1 Department of Biomedical Science and Environmental Biology, College of Life Science, Kaohsiung Medical University, Kaohsiung, 80708, Taiwan; 2 Institute of Biomedical Sciences, Academia Sinica, Taipei, 11529, Taiwan; 3 Division of Nephrology, Chang Gung Memorial Hospital, Kaohsiung Medical Center, Chang Gung University College of Medicine, Kaohsiung, 83301, Taiwan; 4 Institute of Medical Science, College of Medicine, Tzu Chi University, Hualien County, 97004, Taiwan

**Keywords:** Nitric oxide, Rosuvastatin, S-nitrosylation, Proteome, Biotin switch, Endothelial cells

## Abstract

**Background:**

The pleiotropic effects of 3-Hydroxy-3-methylglutaryl coenzyme A reductase inhibitors (statins), which are independent from their cholesterol-lowering action, have been widely recognized in various biological systems. Statins can affect endothelial homeostasis, which is partly modulated by the production of nitric oxide (NO). However, it is unclear how statin/NO-mediated posttranslational S-nitrosylation of endothelial proteins and changes in translational profiles may benefit endothelial integrity. Therefore, it is important to understand the statin/NO-mediated S-nitrosylation in endothelial cells.

**Results:**

Rosuvastatin treatment of human umbilical vein endothelial cells (ECs) enhanced the enzymatic activity of endothelial nitric oxide synthase (eNOS) and the expression of 78 S-nitrosoproteins. Among these S-nitrosoproteins, we identified 17 proteins, including protein disulfide bond isomerase, phospholipase C, transaldolase and heat shock proteins. Furthermore, a hydrophobic Cys66 was determined as the S-nitrosylation site of the mitochondrial HSP70. In addition to the statin-modulated posttranslational S-nitrosylation, changes in the NO-mediated translational proteome were also observed. Seventeen major proteins were significantly upregulated after rosuvastatin treatment. However, 12 of these proteins were downregulated after pretreating ECs with an eNOS inhibitor (L-NAME), which indicated that their expression was modulated by NO.

**Conclusions:**

ECs treated with rosuvastatin increase eNOS activation. The increased NO production is involved in modulating S-nitrosylation and translation of proteins. We provide further evidence of the pleiotropic effect of rosuvastatin on endothelial physiology.

## Background

The 3-hydroxy-3-methylglutaryl coenzyme A (HMG-CoA) reductase inhibitors, commonly referred to as statins, are prescribed to patients to improve serum lipid profiles. The cholesterol-independent pleiotropic effects of statins include improvement of endothelial function, reduction of oxidative stress, prevention of platelet aggregation and stabilization of atherosclerotic plaques [1]. Statins exert a protective effect against plaque inflammation and rupture by the reduction of cyclooxygenase-2 and matrix metallopeptidase-9 expression [2]. Statins also increase the expression of heme oxygenase-1, which is involved in the stress response [3]. Increasing evidence suggests that nitric oxide (NO) plays a role in these statin-mediated pleiotropic effects. Statin treatment increases endothelial nitric oxide synthase (eNOS) expression by enhancing eNOS mRNA stability [4]. Statins promote eNOS activity through the AMP-activated protein kinase (AMPK) and PI3K/Akt pathways [5,6]. This statin-mediated eNOS activation exerts antioxidant effects that decrease reactive oxygen species production [7]. Upregulation of eNOS by statins protects against stroke [8]. Statins induce eNOS activation during hypoxia-induced pulmonary hypertension [9]. Krueppel-like factor-2, a transcription factor responsible for eNOS expression, is upregulated after statin treatment [10].

S-nitrosylation of proteins involves a covalent reaction between NO and a reactive cysteine thiol to form S-nitrosothiol. This cGMP-independent posttranslational modification contributes to various stimuli-mediated cellular signaling [11]. S-nitrosylation can influence protein structure/conformation and enzyme activity. Studies have suggested that S-nitrosylation prevents thiols from irreversible oxidative modification [5,12]. S-nitrosylation has emerged as a fundamental protein modification that is essential for normal cardiovascular function. It is conceivable that NO-mediated S-nitrosylation and protein expression contribute to statin-induced pleiotropic effects.

Rosuvastatin is hydrophilic and possesses the lowest inhibition coefficient (IC50 =5.4 nM) compared with the other statins [13]. Rosuvastatin protects the vasculature by attenuating the release of inflammatory mediators and by suppressing monocyte adhesion. In addition, the activation of c-Jun N-terminal kinases and nuclear factor kappa-light-chain-enhancer of activated B cells is inhibited after rosuvastatin treatment [14]. Mice pretreated with rosuvastatin show increased endothelial NO production and attenuated myocardial necrosis after ischemia/reperfusion [15]. In the clinical setting, patients that switch to rosuvastatin from other statins have significantly lower low-density lipoprotein levels [16].

Considering the rapid progress in proteomics research, it would be interesting to determine the role of NO on rosuvastatin-exerted pleiotropic effects. In the current study, we found that human endothelial cells (ECs) treated with rosuvastatin stimulated eNOS activity with a subsequent increase in S-nitrosylation of endothelial proteins. By using a modified biotin switch approach, we identified the S-nitrosoproteome regulated by rosuvastatin. Moreover, endothelial protein expression was increased after rosuvastatin treatment, but it was suppressed by pretreating ECs with an eNOS inhibitor, indicating that NO modulates rosuvastatin-mediated proteins expression. Our results provide an opportunity to further elucidate the detailed mechanisms by which statin-regulated NO production contributes to lipid-lowering-independent pleiotropic effects.

## Results

### Rosuvastatin activates eNOS and increases protein S-nitrosylation

**Figure 1:**
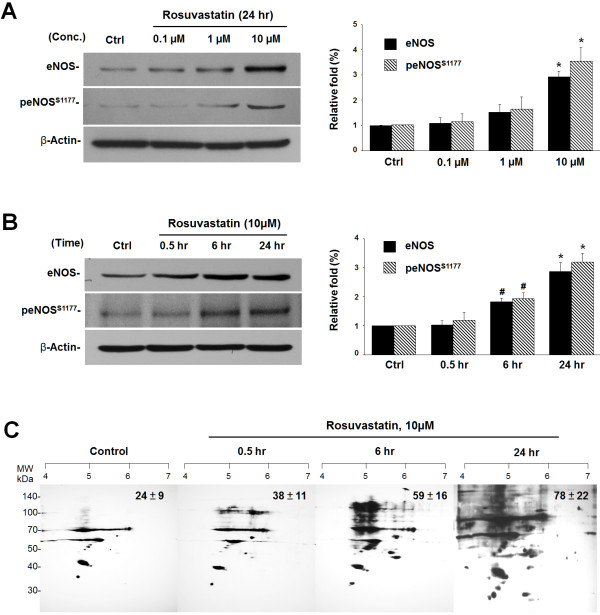
**Rosuvastatin increases eNOS activation and subsequent protein S-nitrosylation in ECs.** (**A**) Protein lysates (40 μg) extracted from ECs treated with rosuvastatin (0.1 μM, 1 μM and 10 μM) for 24 h, or (**B**) rosuvastatin (10 μM) for 0.5, 6, and 24 h were separated by SDS-PAGE and western blotting with antibodies against eNOS (1:3000), phospho-eNOS^S1177^ (peNOS^S1177^, 1:3000) and β-actin (1:5000). The relative fold eNOS and peNOS^S1177^ are shown by mean ± S.E. compared with control treatment. ^**#**^*p* < 0.05, *p < 0.01 from three separate experiments by using Fisher’s LSD. (**C**) One milligram of biotin-switch-derived biotinylated lysate collected from ECs with rosuvastatin (10 μM) treatment for various intervals (0.5, 6 and 24 h) was analyzed by western blot-based 2-DE (streptavidin-HRP, 1:3000). The spot numbers detected on the 2-DE x-ray films are shown as mean ± S.E. from three independent experiments.

In this study, ECs treated with increasing concentrations (0.1, 1 and 10 μM) of rosuvastatin for 24 hours showed an enhanced eNOS expression and a greater phosphorylation of eNOS at ser1177 residue (peNOS^S1177^) compared with controls (Figure 1A). Consistently, rosuvastatin (10 μM) treatment for various time intervals (0.5, 6 and 24 h) also resulted in increased eNOS expression and peNOS^S1177^ compared with controls (Figure 1B). There was a greater than 3-fold increase of eNOS activation after rosuvastatin treatment. By using the modified biotin switch method coupled with western blot-based DTT-free 2-DE, rosuvastatin treatment resulted in a time-dependent increase in S-nitrosylation of protein that occurred at the same time as rosuvastatin-elevated eNOS activation (Figure 1C). The increased amount of S-nitrosoproteins were detected (24 ± 9, 38 ± 11, 59 ± 16 and 78 ± 22) from control and rosuvastatin treatments (0.5, 6 and 24 h).

### Rosuvastatin increases S-nitrosylation in a posttranslational manner

**Figure 2:**
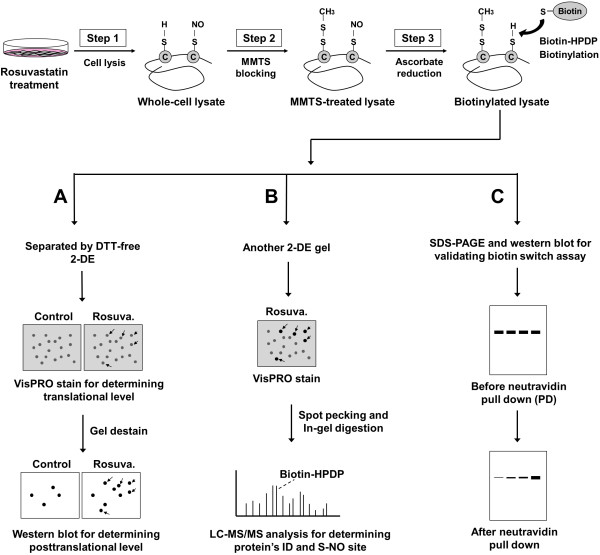
**Biotin switch method to detect protein S-nitrosylation.** As shown in the flowchart, the biotin switch was performed by a three-step reaction. (**A**) The biotinylated lysates were subjected to DTT-free (non-reduced) 2-DE that was stained by VisPRO dye to determine translational levels (total protein). Western blotting was subsequently performed by hybridization with streptavidin-HRP to determine posttranslational S-nitrosylation levels. (**B**) Another separated 2-DE gel was prepared for the pecking of the S-nitrosoproteins and then it was analyzed by mass spectrometry. (**C**) The biotinylated lysates were also applied to a pull-down assay by neutravidin agarose to assure that the identified proteins were biotinylated (i.e. S-nitrosylated).

**Figure 3:**
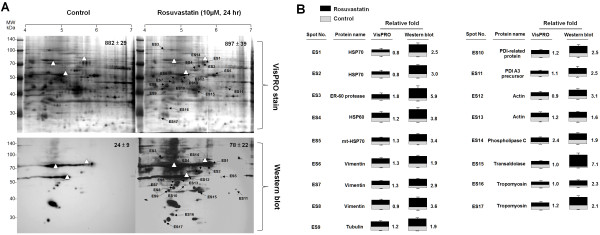
**Rosuvastatin-induced protein S-nitrosylation is a posttranslational event.** (**A**) The 2-DE gels were stained by VisPRO dye to evaluate translational levels. Western blotting was subsequently performed to determine posttranslational levels. Open triangles indicate reference proteins in each gel and x-ray film for confirming the accurate position of each protein spot. Protein spots with increased S-nitrosylation are indicated by arrowheads. (**B**) Rosuvastatin-induced changes at the translational level (column labeled “VisPRO”) and posttranslational S-nitrosylation (column labeled “western blot”) of each S-nitrosoprotein represented as relative fold changes (black bar/gray bar). Statistical data are shown as mean ± S.E. from three separate experiments.

To determine if rosuvastatin-induced S-nitrosylation is a posttranslational event, western blotting and LC-MS/MS were applied (Figure 2a). Briefly, VisPRO staining showed total protein levels and western blotting showed protein modification status. Upon image analysis of western blots, 17 proteins (ES1-ES17) from rosuvastatin-treated ECs showed a significant increase in S-nitrosylation compared with that in control ECs (relative fold ≥1.5, Figure 3A). VisPRO staining showed that the expression of the proteins mentioned above was not affected by rosuvastatin treatment (relative fold between 0.8–1.3), except for proteins at spots ES3 and ES14 (Figure 3B). Our results suggest that increased S-nitrosylation is mainly a posttranslational event.

### Verification of S-nitrosylation of protein and the nitrosylated site

**Figure 4:**
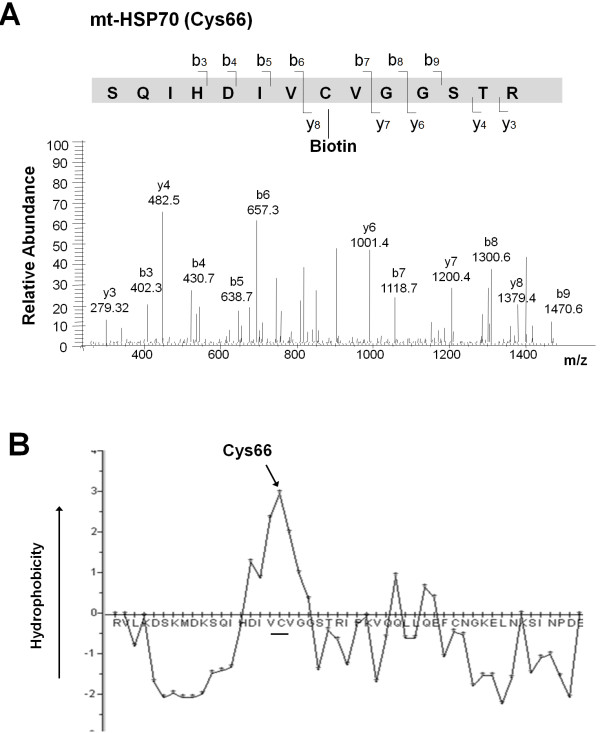
**Cys66 of mitochondrial heat shock protein 70 is the S-nitrosylation site.** (**A**) MS/MS data for peptide fragments from mitochondrial heat shock protein 70 (mt-HSP70) were searched against the NCBInr protein database using a MASCOT in-house algorithm using the precursor ion *m/z* tolerance of 6 ppm. The biotinylated eptides were identified by a mass shift of 428.2 Da. (**B**) The hydrophobicity of the region containing the S-nitrosylated cysteine (Cys66) was predicted by HYDROPHOBICITY PLOT software.

**Figure 5:**
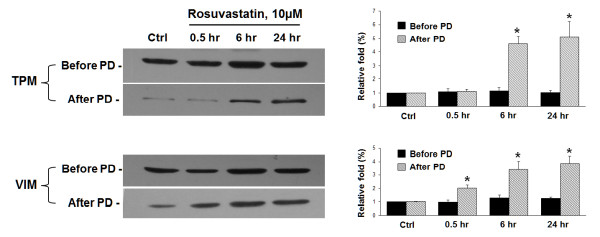
**Rosuvastatin-induced S-nitrosylation of vimentin and tropomyosin.** Forty micrograms of biotinylated lysates derived from the biotin switch method were liquot and separated by SDS-PAGE following western blotting with antibodies against vimentin (VIM) and tropomyosin (TPM). These lysates were assigned as “before pull-down” (PD). The other biotinylated lysates (~500 μg) were incubated with 10 μl neutravidin-agarose to PD biotinylated proteins and assigned as “after PD”. The pulled-down lysates then underwent western blotting with the same antibodies. The relative fold “before PD” and “after PD” changes are shown by mean ± S.E. compared with control treatment. *p < 0.01 from three separate experiments by using Fisher’s LSD.

As shown in Figure 2B, the biotinylated lysate was separated by a 2-DE gel and analyzed by mass spectrometry. Rosuvastatin-induced S-nitrosoproteins were identified that included protein disulfide isomerase-related protein (PDIRP), PDI A3 precursor (PDIA3), HSP60, HSP70, mitochondrial heat shock protein 70 (mt-HSP70) and phospholipase C (Table 1). Further analysis using the MASCOT in-house algorithm against the NCBInr database identified Cys66 as the biotinylated (i.e., S-nitrosylated) site of mt-HSP70 (Figure 4A), and it was located in a hydrophobic region of this protein (Figure 4B). To further verify the S-nitrosoproteins detected by mass spectrometry, the biotinylated lysate was subjected to pull-down assay as described in Figure 2C. Lysates before and after neutravidin pull-down (PD) were analyzed by western blotting using antibodies against tropomyosin (TPM) and vimentin (VIM) (Figure 5). TPM and VIM exhibited a 5.6- and 4.2-fold increase in S-nitrosylation compared with controls.

**Table 1:** S-nitrosylated proteins identified by LC-MS/MS

**Spot number**	**Protein name **^***a)***^	**Accession number **^***b)***^	**Theoretical MW (kDa)/pI**^***c)***^	**Experimental MW (kDa)/pI**^***d)***^	**Sequence coverage (%)**	**MOWSE score**	**Peptides matched**
ES1	HSP70	http://21040386	73.8/6.0	76.3/5.8	47	670	24
ES2	HSP70	http://21040386	73.8/6.0	75.8/5.8	50	717	29
ES3	ER-60 protease	1208427	57.2/5.9	82.5/4.6	26	442	8
ES4	HSP60	31542947	61.0/5.7	63.4/5.6	24	337	10
ES5	mt-HSP70 ^*b)*^	14917005	59.2/5.9	57.2/5.8	34	257	12
ES6	Vimentin	http://340219	53.6/5.0	56.4/4.8	65	998	26
ES7	Vimentin	http://340219	53.6/5.0	56.2/4.7	49	798	21
ES8	Vimentin	http://340219	53.6/5.0	55.8/4.6	55	824	23
ES9	Tubulin	34740335	50.1/4.9	55.0/4.6	32	367	11
ES10	PDI-related protein	1710248	46.2/5.0	56.3/5.0	20	284	7
ES11	PDI A3 precursor	729433	56.9/6.2	55.4/6.4	37	571	11
ES12	Beta-actin	4501885	41.7/5.3	57.2/5.2	61	872	21
ES13	Beta-actin	4501885	41.7/5.3	56.4/5.1	42	605	14
ES14	Phospholipase C-alpha	303618	57.1/6.2	64.2/5.7	11	194	3
ES15	Transaldolase	5803187	37.7/6.4	54.8/5.6	16	269	6
ES16	Tropomyosin 3	55665775	29.1/4.8	34.5/4.7	27	371	6
ES17	Tropomyosin 3	55665775	29.1/4.8	28.6/4.6	28	395	7

a) The function of the protein was obtained via the MASCOT software (www.matrixscience.com) search program by querying the NCBInr database. The parameters were set as follows: peptide mass tolerance: **±** 0.4 Da; allowed missed cleavage: 1.

b) Accession number from the NCBInr database.

c) Protein molecular weight and pI annotated in the database.

d) Protein molecular weight and pI calculated from 2-DE gels.

### Nitric oxide is involved in rosuvastatin-modulated protein expression

**Figure 6:**
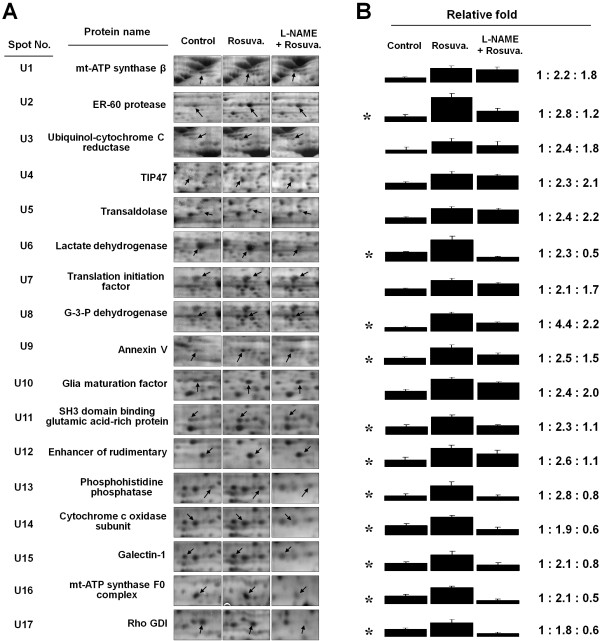
**Rosuvastatin regulates protein expression in a NO-dependent manner.** (**A**) Protein lysates (1 mg) from ECs treated with rosuvastatin (10 μM) alone or with rosuvastatin in the presence of L-NAME (1 mM) for 24 h were subjected to 2-DE. Results are shown by VisPRO staining. Arrows indicate the proteins that were upregulated by rosuvastatin. (**B**) Each asterisk indicates a protein that was upregulated by rosuvastatin but was suppressed by L-NAME co-treatment. The relative fold change in protein level is shown by spot density as mean ± S.E. from three separate experiments.

The translational proteome in ECs after 24 h of rosuvastatin (10 μM) treatment was analyzed by 2-DE (Figure 6A). Seventeen major proteins were upregulated (U1-U17) with a protein intensity greater than 1.5-fold. After mass spectrometric analysis, these proteins were functionally categorized as being involved in lipid metabolism (TIP47), energy regulation (glucose-3-phosphate dehydrogenase, lactate dehydrogenase, transaldolase, cytochrome c oxidase, mt-ATP synthase beta subunit, and F0 complex), angiogenesis (glia maturation factor and galectin-1) and antioxidant-related proteins (glutaredoxin and ubiquinol-cytochrome c reductase) (Table 2). Interestingly, 12 of those 17 statin-upregulated proteins were suppressed when ECs were co-treated with the eNOS inhibitor N^G^-nitro-L-arginine methyl ester (L-NAME, Figure 6B), indicating that NO is involved in rosuvastatin-modulated protein expression in ECs.

**Table 2:** Rosuvastatin-modulated proteins identified by LC-MS/MS

**Spot number**	**Protein name**^***a)***^	**Accession number**^***b)***^	**Theoretical MW (kDa)/pI**^***c)***^	**Experimental MW (kDa)/pI**^***d)***^	**Sequence coverage (%)**	**MOWSE score**	**Peptides matched**
U1	Mitochondrial ATP synthase beta subunit precursor	32189394	51.8/5.0	48.6/5.0	63	1177	22
U2	ER-60 protease	1208427	57.2/5.9	56.5/5.9	54	687	28
U3	Ubiquinol-cytochrome c reductase core I protein	515634	53.2/5.9	46.7/5.7	58	507	23
U4	Cargo selection protein TIP47	3095186	47.2/5.3	45.1/5.3	41	473	15
U5	Transaldolase	5803187	37.7/6.4	36.5/6.1	44	216	16
U6	L-lactate dehydrogenase B	4557032	36.9/5.7	35.7/5.9	40	505	15
U7	Eukaryotic translation initiation factor 3, subunit 2 beta	4503513	36.9/5.4	36.6/5.6	38	273	11
U8	Glyceraldehyde-3-phosphate dehydrogenase	31645	36.2/8.26	36.2/5.5	22	297	6
U9	Annexin V, chain A	157833780	36.0/4.9	35.1/5.1	27	125	11
U10	Glia maturation factor, beta	4758442	16.7/5.2	26.5/5.4	45	208	8
U11	SH3 domain binding glutamic acid-rich protein-like	4506925	12.8/5.2	16.2/5.5	20	91	2
U12	Enhancer of rudimentary homolog	4758302	12.3/5.6	12.8/5.6	17	61	12
U13	Phosphohistidine phosphatase 1 isoform 3	24475861	13.8/5.7	14.4/5.8	26	137	5
U14	Cytochrome c oxidase subunit V precursor	190885499	16.8/6.3	14.2/5.3	22	99	4
U15	Galectin-1	4504981	14.7/5.7	14.5/5.0	38	190	5
U16	ATP synthase, H^+^ transporting, mitochondrial F0 complex, subunit d, isoform a	5453559	18.5/5.2	22.6/5.4	31	127	5
U17	Rho GDP dissociation inhibitor (GDI) beta	56676393	23.0/5.1	26.7/5.2	27	140	7

a) The function of the protein was obtained via the MASCOT software (www.matrixscience.com) search program by querying the NCBInr database. The parameters were set as follows: peptide mass tolerance: **±** 0.4 Da; allowed missed cleavage: 1.

b) Accession number from the NCBInr database.

c) Protein molecular weight and pI annotated in the database.

d) Protein molecular weight and pI calculated from 2-DE gels.

## Discussion

Statins have been widely used as lipid-lowering drugs prescribed to patients with cardiovascular diseases or metabolic disorders. Of particular interest, statins have been shown to increase bioavailability of NO and to protect against vascular inflammation and cardiac cell death [1,3]. Among NO-mediated effects, NO-induced S-nitrosylation of proteins has been recognized as a fundamental posttranslational modification of proteins [5]. This S-nitrosylation of proteins may exert statin-mediated pleiotropic effects in vascular ECs. In the current study, rosuvastatin increased eNOS and activation in a sustained manner (Figure 1), which is consistent with a previous study [15]. The increased S-nitrosylation of proteins was analyzed by a modified biotin switch method as previously described [17]. This approach has an advantage over other reported methodologies [18-20]. Further MS/MS analysis and use of a software algorithm identified the S-nitrosylated site of mt-HSP70 (Cys66) with a protein mass shift of 428.2 Da. To confirm the accuracy of the MS/MS data, the S-nitrosylation of two proteins (TPM and VIM) were verified by their respective antibodies (Figure 5). These S-nitrosylated proteins are heterogeneous in function and may play protective roles for the pleiotropic effects induced by this lipid-lowering drug.

HSPs exert a chaperon function and are involved in protection as well as pathogenesis of various tissues [21]. In our study, three HSPs were identified with characteristics of enhanced S-nitrosylation. HSP60 and HSP70 are involved in the development of atherosclerosis [21]. Mt-HSP70 has been identified as a HSP60 receptor on the cell surface, and it participates in autoantibody-induced endothelial apoptosis [22]. The protective effects of statins on the vascular system may be partially caused by S-nitrosylation of these HSPs due to the known benefit of statins on endothelial integrity. Our previous study demonstrated that ECs under shear flow enhance the S-nitrosylation of HSP70 [23]. S-nitrosylation of Cys597 in HSP90 reduces the ability to activate eNOS [24]. In the present study, S-nitrosylation of Cys66 on the hydrophobic region of mt-HSP70 is consistent with the S-nitrosylation consensus motif that is surrounded by the hydrophobic microenvironment [25]. S-nitrosylation of Cys66 mt-HSP70 may modulate chaperon function as previously suggested [26]. It is unknown whether S-nitrosylation alters HSP properties that contribute to statin-derived pleiotropic effects, and therefore, this warrants further study.

Protein disulfide isomerase (PDI) catalyzes the formation and breakage of disulfide bonds within proteins. S-nitrosylation abrogates PDI-attenuated neurotoxicity [27]. PDI has also been identified as an “NO carrier” that facilitates the introduction of NO from outside the cell via S-nitrosylation/de-nitrosylation [28]. Our previous studies showed that ECs exposed to shear flow or an NO donor increase S-nitrosylation of PDI and related proteins [17,23]. In the present study, PDI-related protein (ES10) and PDI A3 precursor (ES11) were found to be S-nitrosylated in statin-treated ECs. S-nitrosylation of these PDI-related proteins may be physiologically important for maintaining endothelial integrity.

In the current study, three cytoskeletal proteins, vimentin (intermediate filaments), tubulin (microtubules) and actin (microfilaments) were identified with enhanced S-nitrosylation under rosuvastatin treatment. In addition, TPM, which regulates actin movement, was also found to be S-nitrosylated. These proteins are involved in the regulation of cell shape, cell adhesion and migration. Previous studies have indicated that these proteins could be S-nitrosylated under certain conditions [29,30]. It is unclear whether S-nitrosylation of these cytoskeletal proteins affects endothelial migration after statin treatment.

In addition to rosuvastatin-mediated posttranslational S-nitrosylation, we also investigated translational protein levels. Some proteins were shown to be upregulated by rosuvastatin treatment and then suppressed by L-NAME pretreatment, indicating that NO participates in rosuvastatin-induced protein expression. These proteins were functionally grouped into glucose homeostasis (lactate dehydrogenase and glucose-3-phosphate dehydrogenase), energy production (cytochrome c oxidase and mt-ATPase F0 complex), protein folding (ER-60 protease) and anti-inflammation (Rho GDP dissociation inhibitor [GDI]). ER-60 protease (U2) is a cysteine protease that degrades misfolded proteins in the endoplasmic reticulum [31]. Rho-family proteins act as activators of a number of nuclear transcription factors that may reduce NO production and promote inflammatory responses via the Rho-Rho kinase pathway. We also identified a negative regulator of Rho proteins, Rho GDI [3]. Statins have been found to disrupt the interaction between Rho and Rho GDI, and therefore, negatively regulate Rho-family function, which is consistent with the anti-inflammatory effect of rosuvastatin [32].

Our results clearly indicated that rosuvastatin treatment increased S-nitrosylation of proteins and altered protein expression. The detailed mechanisms of the protein expression modulated by rosuvastatin-induced NO remain unclear and need to be further investigated. Nevertheless, these posttranslational S-nitrosoproteomes and translational proteomes provide a basis for further study on the pleiotropic effects of statins in the cardiovascular system.

## Conclusions

Pleiotropic effects of statins have attracted attention from therapeutic and clinical researchers. By using a modified biotin switch methodology, we were able to identify 17 S-nitrosoproteomes regulated by rosuvastatin. In addition, L-NAME treatment confirmed that nitric oxide plays an important role in endothelial protein expression. Our results provide further evidence of the detailed mechanisms of statin/NO-mediated pleiotropic effects.

## Methods

### EC culture

ECs were isolated from the human umbilical cord as described previously [33]. The experimental procedure conformed to the principles outlined in the 1964 Declaration of Helsinki for the use of human tissue or subjects. ECs were cultured in M199 medium supplemented with fetal bovine serum (20%, v/v), streptomycin (100 μg/ml) and penicillin (100 U/ml). ECs were replaced with M199 medium containing 2% (v/v) fetal bovine serum and were incubated overnight prior to rosuvastatin (AstraZeneca, Taipei, Taiwan) treatment. To evaluate whether protein expression was NO-dependent, ECs were co-incubated with an eNOS inhibitor, (L-NAME, 1 mM; Calbiochem) and rosuvastatin (10 μM) for 24 h.

### Cell lysis and protein extraction

ECs after rosuvastatin treatment were washed with cord buffer (0.14 M NaCl, 4 mM KCl, 11 mM glucose, and 10 mM HEPES, pH 7.4), and then lysed with 300 μl lysis buffer (250 mM Hepes, pH 7.7, 1 mM EDTA, 0.1 mM neocuproine and 0.4%,w/v CHAPS). After centrifugation at 10,000 × g for 10 min at 4°C, the supernatant was collected, and the protein concentration of the supernatant was determined with the BCA assay (Thermo Fisher Scientific Inc., USA).

### Two-DE

For 2-DE separation, protein lysates were precipitated with ice-cold acetone at −20°C. After centrifugation, the protein pellets were air-dried and dissolved in sample buffer (9 M urea, 2%, w/v CHAPS,60 mM DL-dithiothreitol [DTT], and 2% v/v IPG solution, pH 4–7, GE Healthcare, USA) at room temperature for 30 min. The denatured proteins were mixed with rehydration buffer (8 M urea, 2% w/v CHAPS, 0.5% v/v IPG solution, pH 4–7, and 30 mM DTT) and soaked into an 18-cm DryStrip (pH 4–7, GE Healthcare) for up to 12 h using an Ettan IPGphor system (GE Healthcare). After isoelectric focusing at an accumulated voltage of 60 kVh, the strip gels were incubated with equilibration buffer (2% w/v SDS, 50 mM Tris–HCl, pH 8.8, 6 M urea, 30% v/v glycerol and 60 mM DTT) for 20 min, and then they were equilibrated with the same buffer containing iodoacetic acid (IAA, 135 mM) for an additional 20 min. The equilibrated isoelectric focusing strip was placed on the top of a vertical SDS-PAGE to separate proteins.

### Biotin-switch

S-nitrosylation was determined by the biotin switch method as previously described by Jaffrey [34]. In brief, the treated ECs were washed with cold buffer (10 mM Hepes, pH 7.4, 0.14 M NaCl, 4 mM KCl and 11 mM glucose). Cell lysates were obtained with lysis buffer (250 mM Hepes, pH 7.7, 1 mM EDTA, 0.1 mM neocuproine, and 0.4% w/v CHAPS). Free thiols in the protein extracts (0.8 mg/ml) were methylated with blocking buffer (225 mM Hepes, pH 7.7, 0.9 mM EDTA, 0.09 mM neocuproine, 2.5% w/v SDS and 20 mM MMTS) at 50°C for 20 min with agitation. MMTS-treated lysate was precipitated with cold acetone and the resulting pellet was resuspended in HENS buffer (250 mM Hepes, pH 7.7, 1 mM EDTA, 0.1 mM neocuproine and 1% w/v SDS). This was followed by addition of HENS buffer containing 4 mM *N*-[6-(biotinamido)hexyl]-3′-(2′-pyridyldithio) propionamide (biotin-HPDP/DMF) mixed with 1 mM sodium ascorbate. The protein lysate:biotin-HPDP mixture was incubated at room temperature for 1 h to allow biotinylation to occur. These mixtures were precipitated with cold acetone to remove excess biotin-HPDP and then stored at −20°C for subsequent studies.

### Detection of biotinylated proteins with non-reduced 2-DE

In the current study, biotinylated lysates derived from the biotin switch were dissolved in the buffers acquired for 2-DE analysis. To prevent the dissociation of labeled biotin molecules, DTT was excluded from those buffers that were used for the translational proteome as described in the above section. Two-DE gels were stained with VisPRO (Visual Protein Biotech, Taipei, Taiwan) to determine protein translational levels, and then western blotting was performed with streptavidin-HRP (1:3000) to determine proteins that were posttranslationally S-nitrosylated. Western blotted membranes were developed with Super Signal West Femto reagent (Thermo Fisher Scientific) and exposed to X-ray film. The X-ray film and the VisPRO-stained gel were scanned by a digital scanner (Microtek, International Inc., Taipei, Taiwan). Results were analyzed by Progenesis Samespots v2.0 software (NonLinear Dynamics, UK). The proteins with increased S-nitrosylation were collected from a separate 2-DE gel with VisPRO staining and were subjected to mass analysis as previously described [17].

### Mass spectrometry analysis

Excised gel slices were digested with trypsin for 4 h at 37°C (In-Gel Tryptic Digestion kit, Thermo Fisher Scientific). The tryptic peptides were desalted on a proteomics C18 column (Mass Solution Ltd., Taiwan) and subjected to mass analysis by CapLC/Q-TOF (Micromass, UK). Mass spectrometry data were searched against the NCBInr database using a MASCOT in-house search program (Matrixscience, UK). Peptides containing a biotinylated cysteine were determined with the mass shift of 428.2 Da. Search parameters were set as follows: mass values, monoisotopic; protein mass, unrestricted; peptide mass tolerance, ± 0.4 Da; fragment mass tolerance, ± 0.4 Da; and maximum missed cleavages, 1. The hydrophobicity of S-nitrosylated cysteine (Cys66) was predicted by HYDROPHOBICITY PLOT software on the website (http://www.bmm.icnet.uk/~offman01/hydro.html).

### Purification of biotinylated protein

The biotinylated proteins (i.e., the S-nitrosoproteins) were pulled down using neutravidin-agarose beads (15 μl/per mg of initiated protein input) in neutralization buffer (20 mM Hepes, pH 7.7, 100 mM NaCl, 1 mM EDTA and 0.5% v/v Triton X-100). The agarose beads were rinsed with washing buffer (20 mM Hepes, pH 7.7, 600 mM NaCl, 1 mM EDTA and 0.5% v/v Triton X-100), and then incubated with elution buffer (20 mM Hepes, pH 7.7, 100 mM NaCl, 1 mM EDTA and 100 mM 2-mercaptoethanol) for 20 min at 37°C, with gentle agitation to release neutravidin-bound proteins. These eluted biotinylated proteins were mixed with SDS-PAGE sample buffer (2% w/v SDS, 50 mM Tris–HCl, pH 6.8, 30% v/v glycerol and 100 mM 2-mercaptoethanol) to perform SDS-PAGE and western blotting to validate S-nitrosylation of protein detected in 2-DE.

## Competing interests

The authors declare that they have no competing interests.

## Authors’ contributions

BH performed the 2-DE experiments and drafted the manuscript. FAL performed the mass spectrometry analysis. CHW carried out the biotin switch assay. BH and DLW read and approved the final manuscript. All authors read and approved the final manuscript.
